# Association between educational attainment and discharge disposition following incident stroke hospitalization: the Atherosclerosis Risk in Communities study

**DOI:** 10.3389/fstro.2026.1847066

**Published:** 2026-07-08

**Authors:** Ning Li, Erin L. Abner, Silvia Koton, Lena Mathews, Kunihiro Matsushita, Anna M. Kucharska-Newton

**Affiliations:** 1 Department of Biostatistics, College of Public Health, University of Kentucky, Lexington, KY, United States; 2 Department of Epidemiology and Environmental Health, College of Public Health, University of Kentucky, Lexington, KY, United States; 3 Division of Cardiology, Johns Hopkins School of Medicine, Baltimore, MD, United States; 4 Department of Epidemiology, Johns Hopkins Bloomberg School of Public Health, Baltimore, MD, United States; 5 Department of Epidemiology, Gillings School of Global Public Health, University of North Carolina at Chapel Hill, Chapel Hill, NC, United States

**Keywords:** discharge disposition, educational attainment, hospitalization, stroke, stroke severity

## Abstract

**Background:**

The relationship between educational attainment and discharge disposition after stroke hospitalization remains unclear. This study examined whether educational attainment is associated with discharge disposition after incident stroke and whether stroke severity modifies this association.

**Methods:**

The study included Atherosclerosis Risk in Communities (ARIC) participants with an incident stroke hospitalization from 1991 to 2020 who were enrolled in fee-for-service (FFS) Medicare at discharge. Discharge disposition was obtained from hospitalization claims. Educational attainment was categorized as “less than high school” vs. “high school or more.” Multivariable logistic regression models were used to estimate associations between educational attainment and discharge disposition, adjusting for age at stroke, sex, race, and study center. Stroke severity was measured using the Stroke Administrative Severity Index (SASI) derived from Medicare discharge diagnosis codes and assessed as an effect modifier.

**Results:**

Among 976 stroke survivors (mean age 75.5 years; 56.6% women; 31.8% Black), the mean hospital stay was 9.3 days, and 30.1% had an intensive care unit (ICU) stay. Overall, 58.9% were discharged home. The median Elixhauser Comorbidity Index was 3, and 55.5% had a SASI score of zero. Compared with those with at least a high-school education, participants with less than high-school education had similar odds of being discharged home (AOR 0.95; 95% CI: 0.7–1.28). Stroke severity did not significantly modify the association.

**Conclusion:**

Educational attainment was not significantly associated with discharge disposition after incident stroke hospitalization in this cohort, although sample size limits the ability to rule out associations.

## Introduction

Stroke is the fifth leading cause of death in the United States and contributes to serious disability (Tsao et al., 2022). Following the acute phase, approximately 50% of stroke survivors are discharged home (Davis et al., 2003; Lynch et al., 2017; Prvu Bettger et al., 2019; Schrage et al., 2023). Age at discharge (Freburger et al., 2011; Tinl et al., 2014; Stein et al., 2015; Mees et al., 2016), home setting, and family support (Koyama et al., 2011; Tanwir et al., 2014; Mees et al., 2016), stroke severity (Stein et al., 2015; Van der Cruyssen et al., 2015), motor and cognitive function (Nguyen et al., 2007; Stein et al., 2015; Mees et al., 2016; Thorpe et al., 2018), and comorbidity burden (Meijer et al., 2003; Béjot et al., 2012; van Almenkerk et al., 2013) have been reported to be associated with discharge disposition. However, the association of socioeconomic factors with discharge disposition for stroke survivors has not been consistently examined. Income and insurance status have been examined as socioeconomic and access-related factors associated with discharge disposition and post-acute rehabilitation placement. Patients with lower income or without private insurance may experience a greater likelihood of discharging to institutional post-acute care or skilled nursing facilities rather than inpatient rehabilitation, but this study does not distinguish discharge home vs. inpatient facility placement (Freburger et al., 2011). Another study, based on stroke patients in Tennessee, examined discharge home vs. inpatient facilities placement, found that Medicare as primary care was associated with higher odds of inpatient facilities discharge (Cho et al., 2017).

Although educational attainment has been considered in prior studies of post-stroke discharge disposition, it has generally been examined as one of the several socioeconomic predictors rather than as the primary exposure of interest (Schrage et al., 2023). A systematic review on predictors of discharge destination included educational attainment among the factors reviewed but concluded that educational attainment had not been studied sufficiently (Mees et al., 2016). Additionally, whether the association differs by race and sex remains unanswered.

Compared with income and insurance status, studying educational attainment is important because it is usually achieved early in adulthood and remains relatively stable throughout life. Importantly, educational attainment can be captured with reasonable accuracy and may serve as an upstream social determinant influencing many downstream factors, including financial resources, health status, wellness, and living conditions among adult stroke patients. Therefore, educational attainment may represent cumulative socioeconomic advantage or disadvantage at the time of hospitalization. In this study, data from the Atherosclerosis Risk in Communities (ARIC) Study cohort were used to examine the association between educational attainment and discharge disposition following incident stroke overall, by race and sex. The study also hypothesized that this association varies according to the level of stroke severity and ICU stay during hospitalization.

## Methods

### Study population

The ARIC study is an ongoing, prospective, community-based cohort study, designed to investigate risk factors for atherosclerosis and cardiovascular disease (The ARIC Investigators, 1989). The study recruited 15,792 men and women aged 45–64 years between 1987 and 1989 from four U.S. communities: Forsyth County, North Carolina; Jackson, Mississippi; the suburbs of Minneapolis, Minnesota; and Washington County, Maryland. The baseline ARIC examination was conducted from 1987 to 1989. Ongoing participant follow-up is conducted through repeat clinic examinations, annual (semiannual since 2012) telephone interviews, and active surveillance of hospitalizations and mortality (Wright et al., 2021).

All ARIC participants who experienced a definite or probable stroke between 1991 and 2020 were eligible for this study. To capture information on discharge disposition, analyses were limited to participants enrolled in FFS Medicare at the time of hospital discharge. This study excluded participants who were discharged to hospice or expired in the hospital, and those with missing data on educational attainment or discharge disposition. This study also excluded participants who self-reported as of a race other than Black or white, as well as Black participants from the Minneapolis and Washington County communities due to a small sample size.

### ARIC Medicare data linkage

Data for ARIC cohort participants were linked with Centers for Medicare & Medicaid Services (CMS) Medicare enrollment data for the years 1991–2020 using a finder file that included participants' Social Security numbers, sex, date of birth, as well as Medicare ID when available (Kucharska-Newton et al., 2016). Information on participant enrollment in Medicare was derived from monthly indicators of enrollment in Parts A and B and Medicaid buy-in, as provided by annual CMS Medicare Beneficiary Summary files.

### Ascertainment of stroke incidence and hospitalization

Stroke events were identified from annual hospitalization records and adjudicated following ARIC study physician review (Rosamond et al., 1999; Koton et al., 2014) and classified as ischemic or hemorrhagic stroke using established criteria (Rosamond et al., 1999). Stroke incidence was defined as the first stroke hospitalization among participants without a baseline self-report of a physician-diagnosed stroke. Analyses were limited to incident short-term stroke hospitalizations identified both in the ARIC records and in the linked CMS Medicare Provider Annual Review (MedPAR) records (Ruban et al., 2020). The International Classification of Diseases, 9th Revision, Clinical Modification (ICD-9-CM) codes 430–438 (Rosamond et al., 1999; Koton et al., 2014) or 10th Revision, Clinical Modification (ICD-10-CM) codes I60–I69 (Woodfield et al., 2015) were used to identify stroke events in MedPAR records.

### Linkage of stroke event with CMS Medicare claims

Participant ID and the date of the stroke event were used to match incident stroke cases identified from ARIC stroke surveillance with stroke-related hospitalizations identified from CMS MedPAR records. In ARIC stroke surveillance, the date of the stroke event was considered as the date of incidence, while in the MedPAR record, the study utilized the admission date as the date of the event. Following identification of participants with a perfect match based on the unique ID and date of hospitalization, the study performed a “fuzzy match” in which the stroke hospitalization dates matched within 3 days, 1 week, 1 month, or 1 year (Ruban et al., 2020; Kucharska-Newton et al., 2023).

### Ascertainment of educational attainment

Self-reported educational attainment among ARIC participants was obtained at baseline when participants were 45–64 years of age and their education was likely completed. Educational attainment was categorized as “less than high school” (completed years of education < 12) and “high school or more” (completed years of education ≥12).

### Identification of discharge disposition status

Information on discharge disposition was obtained from MedPAR inpatient records and categorized as either “discharge to home” or “discharge to rehabilitation.” In this study, “discharge to home” refers to being discharged to home with or without home health, while “discharge to rehabilitation” was defined as being discharged to intermediate care facilities (ICFs), skilled nursing facility (SNF), inpatient rehabilitation facility (IRF), or other short- or long-term inpatient care facilities. Cases with inpatient death and those with discharge to hospice were excluded from analyses.

### Variables and analysis model design

The multivariable logistic regression models were adjusted for a minimally sufficient set of confounders, including age at stroke hospitalization, self-reported sex and race, and the ARIC study field center (Supplementary Figure 1). To account for confounding of the race variable by study center, the race and study field center were combined into a single variable comprising the following groups: Washington County white participants, Minneapolis white participants, Jackson Black participants, Forsyth County Black participants, and Forsyth County white participants (Mathews et al., 2022).

Covariates plausibly lying in the pathway from educational attainment to discharge disposition, such as body mass index (BMI), physical activity, stroke severity, comorbidities, length of stay (LOS), marital status, ICU stay and income, were not included in analytical models. However, these factors were summarized descriptively to characterize the study cohort. BMI was calculated as weight in kilograms divided by height in meters squared and was obtained from the closest ARIC visit preceding the incident stroke. Smoking status (current, former, never) was defined as the status proximally preceding the incident stroke. Physical activity, assessed with a modified Baecke Physical Activity Questionnaire (Baecke et al., 1982), was represented by average weekly minutes of all sport activities that were collected from ARIC visits close to the incident stroke and treated as a continuous variable. Marital status was collected from annual follow-up in the year of incident stroke or the closest year before the incident stroke hospitalization. The study created a dichotomous variable to represent marital status: married or single (including never married, divorced, separated, or widowed) (Kucharska-Newton et al., 2011). Median family income was collected from the ARIC baseline visit and classified into three levels: < $15,999, $16,000 to 34,999, ≥$35,000 based on previous ARIC studies (Kucharska-Newton et al., 2011). Comorbidity burden was defined using the Elixhauser Comorbidity Index, which includes 31 factors (Elixhauser et al., 1998; Quan et al., 2005). The study created indicators for comorbid conditions based on ICD-9-CM and ICD-10-CM diagnostic codes found at any position in the MedPAR records. Information on LOS was obtained from the MedPAR records. ICU stay, including general, surgical, medical, psychiatric, intermediate, and other intensive care stays, was identified using the MedPAR ICU stay indicator.

To test robustness, we used inverse probability of treatment weighting (IPTW) based on a propensity score model, which created a weighted pseudo-population with confounders balanced across educational attainment groups. This approach estimates the marginal association between educational attainment and discharge disposition. Furthermore, a sensitivity analysis was performed by adding the year of stroke incidence to reduce potential temporal trends due to changes in stroke care and discharge practices over time.

### Stroke severity score

The study utilized the Stroke Administrative Severity Index (SASI) as a measurement of stroke severity (Osborn et al., 2014; Simpson et al., 2018). SASI is a post-discharge validated measure to assess stroke severity. It is intended for use in settings where clinical severity scales, such as the National Institutes of Health Stroke Scale (NIHSS), are unavailable (Simpson et al., 2018). The study derived SASI scores from the discharge diagnosis and procedure code of MedPAR according to the published procedure (Simpson et al., 2018) (Supplementary Table 1). The parameters included aphasia, coma, dysarthria and/or dysphagia, hemiplegia or monoplegia, neglect, nutritional infusion, and tracheostomy and/or ventilation. The intercept in the SASI predictive regression model was omitted from the score calculation, as specified in the published formula (Simpson et al., 2018). In the present study, the SASI score was classified into three groups: mild (score = 0), moderate (score = 1–6), and severe (score ≥ 7) (Simpson et al., 2018).

The formula is shown below:

SASI score = 4^*^(aphasia) + 23^*^(coma) + 2^*^(dysarthria and/or dysphagia) + 6^*^(hemiplegia or monoplegia) + 5^*^(neglect) + 6^*^(nutritional infusion) + 10^*^(tracheostomy and/or ventilation).

### Statistical analysis

Participant characteristics were summarized overall and across educational attainment categories using the chi-square test for categorical variables and *t*-tests and Wilcoxon rank-sum tests for continuous variables. Continuous variables were expressed as means with standard deviations (SDs), except the Elixhauser Comorbidity Index, which was reported as median and interquartile range (IQR). Categorical variables were presented as frequencies and percentages.

In the primary analysis, the overall association between educational attainment and discharge disposition after adjusting for baseline confounders was estimated. Multivariable logistic regression models were fitted to estimate the total effect. Adjusted odds ratios (AORs) and 95% confidence intervals (CIs) were calculated for being discharged to home vs. being discharged to rehabilitation. The study then examined whether this association varied across clinically relevant subgroups by incorporating interaction terms into the model. This approach enabled us to evaluate potential effect modification while preserving the primary analysis as an estimate of the overall association in the study population. The study assessed interaction terms to evaluate whether stroke severity or ICU stay modified the association between educational attainment and discharge disposition.

In all analyses, a two-sided *p*-value of < 0.05 was considered statistically significant. All analyses were performed using SAS 9.4^®^ (SAS Institute Inc., Cary, North Carolina).

## Results

### Baseline characteristics

**Figure 1:**
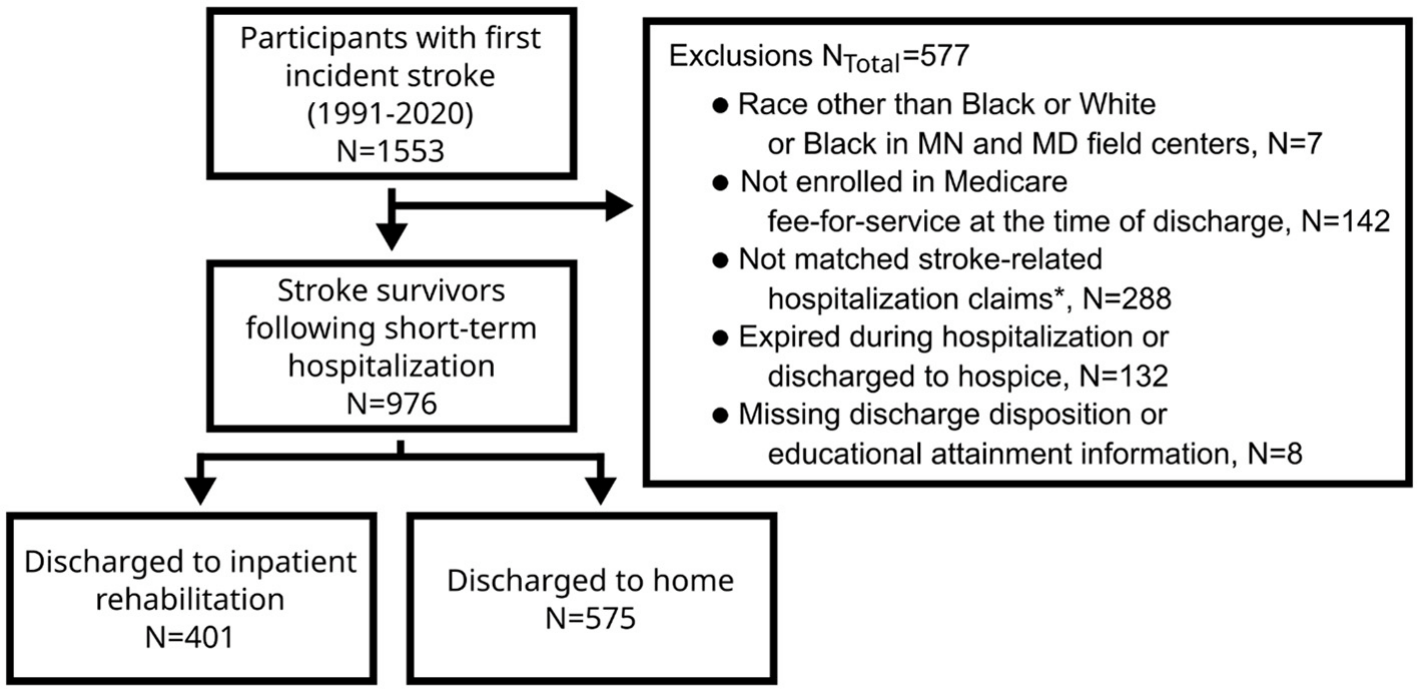
Study cohort flowchart. MN, Minnesota; MD, Maryland. *Participants with incident stroke and Medicare fee-for-service were matched to inpatient claims by ID and the admission date. We included records with exact or fuzzy matches with 3 days, 7 days, 1 month, or 1 year. Participants were excluded if the date of the incident stroke did not match the date of hospitalization admission in this criterion.

A total of 1,553 ARIC participants with an incident stroke were identified through ARIC surveillance of hospitalizations for the years 1991–2020. In total, 577 participants were excluded using the following criteria: race other than Black or white, Black race in the Minneapolis, MN and Washington County, MD field centers (*n* = 7); not enrolled in Medicare Part A (*n* = 142); unmatched hospitalization records (*n* = 288); expired during hospitalization or discharged to hospice (*n* = 132); missing exposure or outcome data (*n* = 8) (Figure 1).

Among 976 stroke survivors (mean age 75.5 years (SD: 7.4), 56.6% women, 31.8% Black), 575 (58.9%) were discharged home, while 401 (41.1%) were discharged to inpatient rehabilitation settings (Figure 1). A total of 30.1% participants experienced an ICU stay. The average LOS was 9.3 days (SD: 10.4). The median of the Elixhauser comorbidity index score was 3 (IQR: 3) (Table 1).

**Table 1:** Characteristics of stroke survivors overall and by educational attainment: the atherosclerosis risk in communities study 1991–2020.

Parameter	All, *N* = 976	Educational Attainment		*P*-value^*^
		<High school	High school or more	
		*N* = 304	*N* = 672	
Age at time of incident stroke, years, mean (SD)	75.45 (7.42)	74.28 (7.81)	75.97 (7.19)	0.001
Women, *n* (%)	552 (56.56)	177 (58.22)	375 (55.8)	0.48
Black race, *n* (%)	310 (31.76)	160 (52.63)	150 (22.32)	< 0.0001
ARIC study field center, *n* (%)				
Forsyth, NC	224 (22.95)	61 (20.07)	163 (24.26)	< 0.0001
Jackson, MS	274 (28.07)	145 (47.7)	129 (19.2)	
Minneapolis, MN	211 (21.62)	16 (5.26)	195 (29.02)	
Washington, MD	267 (27.36)	82 (26.97)	185 (27.53)	
Married, *n* (%)	519 (54.29)	131 (44.26)	388 (58.79)	< 0.0001
ICU stay, *n* (%)	294 (30.12)	83 (27.3)	211 (31.4)	0.2
LOS, days, mean (SD)	9.33 (10.35)	10.26 (11.21)	8.91 (9.91)	0.005
BMI, kg/m^2^, mean (SD)	29.3 (5.77)	30.3 (6.29)	28.84 (5.46)	0.0015
Smoking, *n* (%)	269 (30.36)	96 (35.96)	173 (27.95)	0.02
Physical activity, mins, mean (SD)	135.95 (166.31)	88.69 (137.56)	157.25 (173.7)	< 0.0001
Elixhauser Comorbidity Index,				0.53
Median (IQR)	3 (3)	4 (3)	3 (3)	
(25th−75th percentiles)	(2-5)	(2-5)	(2-5)	
Stroke severity^a^, *n* (%)				0.07
Mild (SASI score = 0)	542 (55.53)	185 (60.86)	357 (53.13)	
Moderate (SASI score: 1–6)	272 (27.87)	72 (23.68)	200 (29.76)	
Severe (SASI score ≥7)	162 (16.6)	47 (15.46)	115 (17.11)	
Total family income^b^, *n* (%)				
< $15,999	304 (31.57)	181 (59.93)	123 (18.61)	< 0.0001
$16,000–34,999	307 (31.88)	86 (28.48)	221 (33.43)	
≥$35,000	352 (36.55)	35 (11.59)	317 (47.96)	

ICU, Intensive care unit; LOS, length of stay; BMI, Body Mass Index; SASI, Stroke Administrative Severity Index; SD, Standard deviation; IQR, interquartile range.

^a^Stroke severity: SASI score based on CMS Medicare claims; range from 0 to 33. Score was developed from multivariable logistic regression model with 7 stroke severity predictors (aphasia, coma, dysarthria and/or dysphagia, hemiplegia or monoplegia, neglect, nutritional infusion, tracheostomy and/or ventilation)

^b^Total family income was collected at the ARIC baseline visit. ^*^P-value from chi-square test for categorical variables and t-test or Wilcoxon rank-sum test for continuous variables.

Compared with participants with at least a high school education, those with less than high school education were slightly younger (74.3 vs. 76.0 years; *P* = 0.001), more often Black (52.6% vs. 22.3%; *P* < 0.0001 and had a longer hospital LOS (10.3 vs. 8.9 days; *P* = 0.005). Use of the ICU was similar across educational attainment groups (27.3% vs. 31.4%; *P* = 0.20) (Table 1).

The SASI stroke severity score, range 0–33 (mean = 3.1, SD = 4.4) (Supplementary Figure 2), was lower among stroke survivors discharged home (mean = 1.8, SD = 3.2) as compared to those discharged to rehabilitation (mean = 5.0, SD = 5.1). Overall, 55.5% of stroke survivors were identified with mild strokes (SASI score = 0) but only 16.6% of stroke survivors were identified as having experienced a severe stroke (SASI Score ≥ 7) (Supplementary Figure 2).

### Association between educational attainment and discharge disposition

After adjustment for age at time of incident stroke, sex, race, and study field center, and the year of stroke incidence, the AORs of discharge to home was 0.95 (95% CI: 0.7–1.28) among those with less than high school education compared to high school or more education (Table 2). No significant differences were observed by race or sex in the association between educational attainment and discharge disposition. Among participants with less than a high school education, compared to those with a high school education or higher, the adjusted odds of being discharged home were 0.86 (95% CI: 0.58–1.28) for white participants and 1.08 (95% CI: 0.69–1.71) for Black participants. Similar patterns were observed across sex: the odds ratio was 0.84 (95% CI: 0.57–1.24) for women and 1.11 (95% CI: 0.7–1.79) for men (Table 2).

**Table 2:** Association of educational attainment with discharge disposition following incident stroke overall.

Characteristic	Discharge to home vs. rehabilitation			
	Overall^a^			
	Home, *N* = 575	Rehabilitation, *N* = 401	AOR	95% CI
Overall				
< High school	178	126	0.95	0.7–1.28
High school or more	397	257	Reference	
Race^b^						
White	*N* = 391	*N* = 257		
< High school	81	63	0.86	0.58–1.28
High school or more	310	212	Reference	
Black	*N* = 184	*N* = 126	
< High school	97	63	1.08	0.69–1.71
High school or more	87	63	Reference	
Sex^c^						
Men	*N* = 261	*N* = 163	
< High school	82	45	1.11	0.70–1.79
High school or more	179	118	Reference	
Women	*N* = 314	*N* = 238	
< High school	96	81	0.84	0.57–1.24
High school or more	218	157	Reference	

^a^Model adjusted for age at the time of incident stroke, sex, and race^*^center.

^b^Model adjusted by the age at the time of incident stroke, sex and center.

^c^Model adjusted by the age at the time of incident stroke, and race^*^center.

AOR, adjusted odds ratio.

The association between educational attainment and discharge disposition remained non-significant in analyses stratified by stroke severity. Among participants with mild stroke, the AORs of discharge to home was 0.87 (95% CI: 0.58–1.32) for those with less than high school education, compared to those with high school or more. Education remained non-significantly associated with discharge disposition among those with moderate strokes (AOR: 0.67, 95% CI: 0.38–1.17) and among those with severe strokes (AOR: 1.38, 95% CI: 0.61–3.12) (Table 3).

**Table 3:** Effect measure modification by stroke severity and ICU stay of the association of educational attainment with discharge disposition following incident stroke hospitalization.

	Stroke severity^a^		
	Home	Rehabilitation^*^	AOR (95% CI)
Mild stroke			
< High school	131	54	0.87 (0.58–1.32)
High school or more	260	97	Reference
Moderate stroke			
< High school	35	37	0.67 (0.38–1.17)
High school or more	115	85	Reference
Severe stroke			
< High school	12	35	1.38 (0.61–3.12)
High school or more	22	93	Reference
ICD Stay^b^			
**Admitted to ICU**			
< High school	35	48	0.91 (0.54–1.56)
High school or more	91	120	Reference
**Not admitted to ICU**			
< High school	143	78	0.92 (0.64–1.32)
High school or more	306	155	Reference

^a^Estimates were calculated from adjusted model, including educational attainment, stroke severity, educational attainment ^*^stroke severity, age at the time of incident stroke, sex, race^*^center.

^b^Estimates were calculated from adjusted model, including educational attainment, ICU stay, educational attainment ^*^ ICU stay, age at the time of incident stroke, sex, race^*^center.

^*^Discharge disposition: rehabilitation (reference)

AOR, adjusted odds ratio.

Similarly, ICU stay did not significantly modify the association of educational attainment and discharge disposition. For those not admitted to the ICU, the AORs for discharge to home was 0.91 (95% CI: 0.54–1.56) for those with less than high school compared to those with high school or more. Meanwhile, among stroke survivors admitted to the ICU, the AORs for discharge to home was 0.92 (95% CI: 0.64–1.32) for those with less than high school compared to those with high school or more (Table 3).

### Sensitivity analyses

Using the IPTW approach, the odds ratio of being discharged to home was 0.98 (95% CI: 0.92–1.08) for less than high school education was observed compared to high school or more (Supplementary Table 2). Findings from analyses stratified by stroke severity and ICU admission were consistent with the main results, showing no significant association between educational attainment and discharge disposition (see Supplementary Tables 3, 4). After adding the year of stroke incidence as a temporal adjustment variable for accounting for secular trends over the study period, the results of the analysis suggest no change in the effect estimates (see Supplementary Table 5).

## Discussion

Among 976 ARIC participants enrolled in FFS Medicare who were discharged following an incident stroke event, 58.9% were discharged home. The study observed no significant association between educational attainment and discharge disposition after stroke hospitalization. Furthermore, the study did not observe any modification of the association between educational attainment and discharge disposition by race, sex, stroke severity, or ICU stay. However, this finding should be interpreted cautiously given the modest sample size, limited clinical factors, stroke severity index misclassification, and caregiver availability and post-acute care eligibility.

The literature on the association of socioeconomic factors with stroke discharge disposition is conflicting. Using State Inpatient Databases linked to US Census data, Freburger et al. (2011) reported that stroke patients with low incomes were more likely to be discharged to institutions rather than returning home. In a separate analysis of admission data from a single hospital, Nguyen et al. observed that stroke survivors residing in high-poverty geographical areas were more likely to be discharged home (Nguyen et al., 2007). Gregory and Han (2009) using data from the North Carolina Hospital Discharge Database, found that higher county-level poverty was associated with a greater likelihood of discharge from inpatient rehabilitation. A recent cohort study in Canada found that material deprivation was not associated with discharge disposition after adjusting for baseline characteristics (MacDonald et al., 2022). A discharge-focused review by Chevalley et al. on socio-environmental predictive factors for discharge destination after inpatient rehabilitation in stroke patients. It pooled evidence for factors such as living with others, support at home, marital status, and pre-stroke living situation (Chevalley et al., 2022). Individual and neighborhood socioeconomic factors may represent important upstream determinants, as they likely influence the availability of material, social, and caregiving resources that shape discharge disposition.

The findings of this study contribute to the literature by evaluating educational attainment as the primary socioeconomic exposure in relation to discharge disposition after incident stroke hospitalization. Prior studies of stroke discharge disposition have more often focused on income, insurance status, or community-level socioeconomic indicators. Less is known about whether education itself is associated with discharge disposition. Therefore, this study addresses an important gap by examining whether this stable upstream social determinant is related to discharge disposition among older stroke survivors. However, the lack of a statistically significant association should be interpreted cautiously. Discharge disposition is a complex outcome influenced by multiple patient-, provider-, facility-, and system-level factors. Clinical factors such as stroke severity, functional impairment, cognitive status, rehabilitation eligibility, caregiver availability, home environment, and local availability of post-acute care services may strongly shape discharge decisions (Nguyen et al., 2007; Koyama et al., 2011; Béjot et al., 2012; Tanwir et al., 2014; Stein et al., 2015; Van der Cruyssen et al., 2015; Mees et al., 2016; Thorpe et al., 2018). Some of these factors were unavailable in this study, raising the possibility of unmeasured confounding.

In addition, the modest sample size may have limited statistical power, especially for subgroup and interaction analyses. Therefore, findings of this study should not be interpreted as evidence that educational attainment has no relationship with discharge disposition. Rather, they suggest that, in this cohort and after adjustment for available covariates, strong evidence of an independent association was not found. Larger studies with more detailed clinical, functional, caregiver, and facility-level information are needed to better clarify whether and how educational attainment influences discharge planning and post-acute care placement after stroke.

This study focuses on the discharge disposition as the outcome because it is a clinically meaningful marker of the transition from acute- to post-acute care (Winstein et al., 2016). Discharge disposition is also policy relevant since it reflects access to different intensities of post-acute care that have been associated with downstream outcomes, such as improvement in physical functioning, and the risk of readmission and mortality (Chovanec et al., 2021). The discharge measure in the study did not distinguish between discharge to the home with or without home health, an important aspect of post-acute care.

Several limitations should be acknowledged. First, because MA claims were not available until 2015, this study was restricted to FFS Medicare beneficiaries. This restriction may introduce selection bias as Black and Hispanic beneficiaries, and those with lower income or lower educational attainment, are more likely to enroll in Medicare Advantage (MA) or select plans with supplemental benefits (Aggarwal et al., 2022). Prior studies also showed that MA and FFS differ in utilization and post-acute care patterns. Enrollment in MA plans as compared to enrollment in FFS Medicare is often associated with fewer hospitalizations, shorter lengths of stay, and lower post-acute care intensity (Agarwal et al., 2021). In this study cohort, the proportion of participants with “less than high school” educational attainment was 4% greater among participants enrolled in MA as compared to those enrolled in FFS Medicare. The analytical sample of this study may have therefore slightly underrepresented participants with lower educational attainment status. If these participants were also more likely to experience facility discharge or different post-acute care placement patterns, restricting the sample to FFS beneficiaries could have biased estimates toward null, although the direction and magnitude of bias remain uncertain. Second, discharge decisions among participants enrolled in Medicare FFS or other insurance coverage may follow standardized protocols that prioritize clinical criteria and bed availability over socioeconomic position, potentially minimizing socioeconomic position-related disparities. Third, educational attainment captures only one dimension of socioeconomic position, with several key aspects not directly addressed, including financial and material resources, health insurance, access to care, neighborhood, and social support resources that may influence discharge planning and access to post-acute care. Fourth, the sample size, particularly in stratified and interaction models, may have been insufficient to detect modest associations, limiting the statistical power to observe subtle differences in effect estimates. Fifth, the use of the Elixhauser comorbidities index is vulnerable to coding variation present in administrative data. Sixth, limitations in measuring stroke severity that are based on the claims data rather than neurological examination may have reduced the ability of this study to fully account for clinical need at discharge. Last, the findings of the study should be interpreted in light of the ARIC study population, which included middle-aged and older adults from selected U.S. communities. Thus, the results may not be fully generalizable to younger stroke survivors or to the broader U.S. population.

## Conclusion

In conclusion, the study found that educational attainment was not significantly associated with discharge disposition following incident stroke hospitalization among ARIC participants. And there was no evidence in this study that stroke severity modified the association of educational attainment with discharge disposition, although this analysis may have been underpowered.

## Data Availability

In accordance with the Data Use Agreement between the ARIC Study and the Centers for Medicare and Medicaid Services (CMS), the CMS Medicare claims information used in this study cannot be shared with other investigators. Further requests should be directed to the corresponding author.
