# Supplementary material for: Association between educational attainment and discharge disposition following incident stroke hospitalization: the Atherosclerosis Risk in Communities study
Source: Front Stroke. 2026 Jul 8;5:1847066. doi: 10.3389/fstro.2026.1847066 (PMC13388064; doi:10.3389/fstro.2026.1847066)
Supplement: Supplementary file 1 [file Supplementary_file_1.docx]

***Supplementary Materials***

**Association Between Educational Attainment and Discharge Disposition Following Incident Stroke Hospitalization: The Atherosclerosis Risk in Communities (ARIC) Study**

Ning Li, Erin L Abner, Silvia Koton, Lena Mathews, Kunihiro Matsushita, Anna M Kucharska-Newton

**Supplementary Figure 1.** Directional Acyclic Graph for the Association between Educational Attainment and Discharge Disposition

**
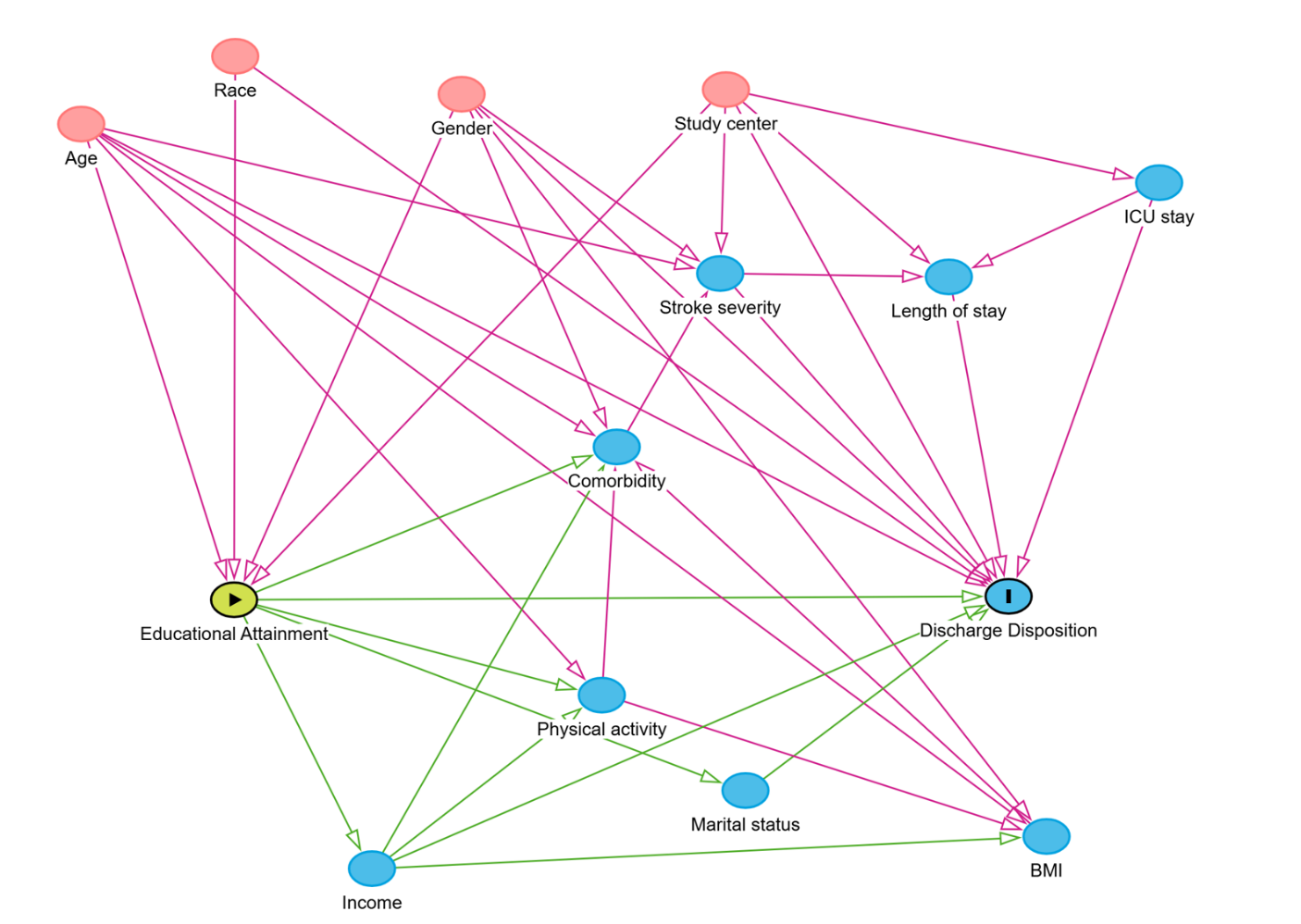
**

**Supplementary Figure 2.** Distribution of Stroke Administrative Severity Index Score Overall and By Discharge Disposition.

**
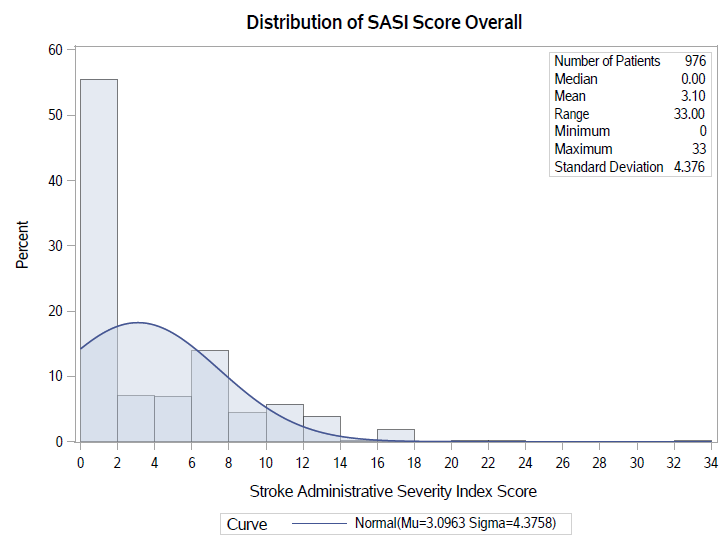

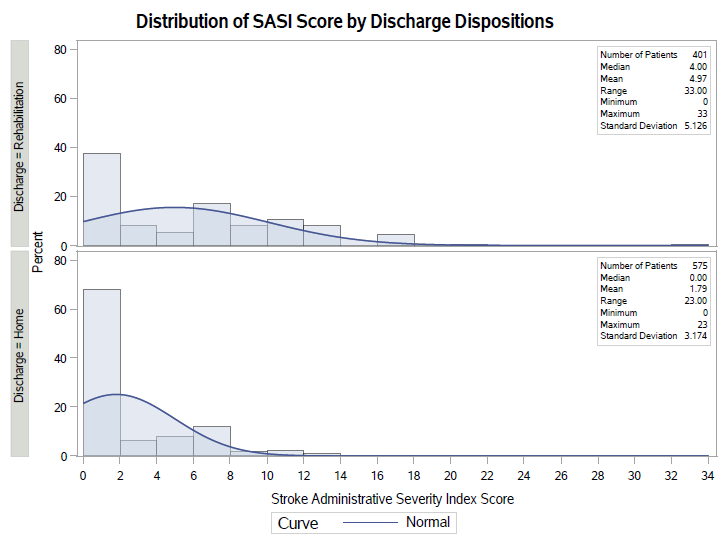
**

**Supplementary Table 1.** Stroke Administrative Severity Index (SASI) Indicators and ICD-CM Codes.

| **Indicators** | **Score^#^** | **ICD-CM diagnosis and procedure code^*^** |
| --- | --- | --- |
| Aphasia | 4 | **ICD-9-CM:** 784.3, 438.11  **ICD-10-CM:** R47.0, R47.01 |
| Coma | 23 | **ICD-9-CM:** 780.01,780.03  **ICD-10-CM:** R40.3, R40.2 |
| Dysarthria and/or dysphagia | 2 | **ICD-9-CM:** 438.13, 438.82, 784.51, 787.2  **ICD-10-CM:** R13.0, R13.10, R13.19, R47.0, R47.02, R47.01, I69.920-I69.922, I69.020-I69.022, I69.320-I69.322, I69.091, I69.191, I69.291, I69.391, I69.891, I69.991 |
| Hemiplegia or monoplegia | 6 | **ICD-9-CM:** 342.90, 438.2, 438.3, 438.4, 438.5  **ICD-10-CM:** G81.90- G81.94, I69.05, I69.15, I69.25, I69.35, I69.85, I69.95 |
| Nutritional infusion | 5 | **ICD-9-CM:** 99.15  **ICD-10-CM:** 3E0336Z, 3E0436Z, 3E0536Z, 3E0636Z |
| Neglect | 6 | **ICD-9-CM:** 781.8  **ICD-10-CM:** R41.4 |
| Tracheostomy and/or ventilation | 10 | **ICD-9-CM:** 31.1, 31.29, 93.90, 96.04, 96.70, 96.7, 96.71, 96.72  **ICD-10-CM:** Z93.0, J95.01, J95.02, J95.850, 0B110F4, 0B110Z4, 0B113F4, 0B113Z4, 0B113Z4, 0B114F4, 0B114Z4, 0BH17EZ, 0BH17EZ, 0BH18EZ, 5A1935Z, 5A1945Z, 5A1955Z, 5A09357, 5A09357, 5A09457, 5A09557 |
| ^#^Score=coefficient from parsimonious multiple logistic regression multiple by 10 and rounded to nearest integer.  ^*^ICD-9-CM and cross-walked ICD-10-CM diagnosis and procedure codes. | | |

**Supplementary Table 2.** IPTW Adjusted Odds Ratio for the Association between Educational Attainment and Discharge Disposition Overall.

|  | **AOR** | **95% CI** |
| --- | --- | --- |
| **< high school** | 0.98 | 0.92-1.08 |
| **High school or more** | **reference** | |
| Outcome: discharge disposition: home vs. rehabilitation (reference)  IPTW: inverse probability of treatment weights. | | |

**Supplementary Table 3.** IPTW Adjusted Odds Ratio Stratified by Stroke Severity for the Association between Educational Attainment and Discharge Disposition Stratified by Stroke Severity.

|  | **Mild Stroke** | **Moderate Stroke** | **Severe Stroke** |
| --- | --- | --- | --- |
|  | **AOR**  **(95% CI)** | **AOR**  **(95% CI)** | **AOR**  **(95% CI)** |
| **< high school** | 0.98  (0.91-1.07) | 1.18  (1.00-1.39) | 0.98  (0.84-1.15) |
| **High school or more** | **reference** | | |
| Outcome: discharge disposition: home vs. rehabilitation (reference)  IPTW: inverse probability of treatment weights. | | | |

**Supplementary Table 4.** IPTW Adjusted Odds Ratio Stratified by Stroke Severity for the Association between Educational Attainment and Discharge Disposition Stratified by Stroke Severity.

|  | **Admitted to ICU** | **No admitted to ICU** |
| --- | --- | --- |
|  | **AOR**  **(95% CI)** | **AOR**  **(95% CI)** |
| **< high school** | 1.02  (0.87-1.19) | 1.01  (0.92-1.09) |
| **High school or more** | **reference** | |
| Outcome: discharge disposition: home vs. rehabilitation (reference)  IPTW: inverse probability of treatment weights. | | |

**Supplementary Table 5.** **Association of Educational Attainment with Discharge Disposition Following Incident Stroke Overall with additional temporary variable: the year of stroke incidence.**

|  | **Discharge to Home vs. Rehabilitation** | | | | |
| --- | --- | --- | --- | --- | --- |
|  | **Overall^1^** | | | | |
|  | **Home**  **N=575** | **Rehabilitation**  **N=401** | **AOR** | | **95% CI** |
| **Overall** |  | | | | |
| < High school | 178 | 126 | 0.92 | | 0.68-1.25 |
| High school or more | 397 | 257 | reference | | |
|  | **Race^2^** | | | | |
| **White** | **N=391** | **N=257** |  |  | |
| < High school | 81 | 63 | 0.87 | 0.58-1.30 | |
| High school or more | 310 | 212 | reference | | |
| **Black** | **N=184** | **N=126** |  | | |
| < High school | 97 | 63 | 0.98 | 0.51-1.56 | |
| High school or more | 87 | 63 | reference | | |
|  | **Gender^3^** | | | | |
| **Men** | **N=261** | **N=163** |  | | |
| < High school | 82 | 45 | 1.08 | 0.67-1.73 | |
| High school or more | 179 | 118 | reference | | |
| **Women:** | **N=314** | **N=238** |  | | |
| < High school | 96 | 81 | 0.82 | 0.55-1.23 | |
| High school or more | 218 | 157 | reference | | |
| **Note:**  ^1^: model adjusted for age at the time of incident stroke, gender, race*center, and year of stroke incidence.  ^2^: model adjusted by the age at the time of incident stroke, gender, center, and year of stroke incidence  ^3^: model adjusted by the age at the time of incident stroke, race*center, and year of stroke incidence.  **AOR:** adjusted odds ratio | | | | | |
